# Employing machine learning for enhanced abdominal fat prediction in cavitation post-treatment

**DOI:** 10.1038/s41598-024-60387-x

**Published:** 2024-05-14

**Authors:** Doaa A. Abdel Hady, Omar M. Mabrouk, Tarek Abd El-Hafeez

**Affiliations:** 1 Department of Physical Therapy for Women’s Health, Faculty of Physiotherapy, Deraya University, EL-Minia, Egypt; 2 MSK Sonographer, Physical Therapy for Basic Science, Deraya University, EL-Minia, Egypt; 3 Department of Computer Science, Faculty of Science, Minia University, EL-Minia, Egypt; 4 Computer Science Unit, Deraya University, EL-Minia, Egypt

**Keywords:** Abdominal fat, Cavitation’s impact, Fat prediction, Hyperopt, Optuna regressor, Health care, Computer science, Information technology, Scientific data, Statistics

## Abstract

This study investigates the application of cavitation in non-invasive abdominal fat reduction and body contouring, a topic of considerable interest in the medical and aesthetic fields. We explore the potential of cavitation to alter abdominal fat composition and delve into the optimization of fat prediction models using advanced hyperparameter optimization techniques, Hyperopt and Optuna. Our objective is to enhance the predictive accuracy of abdominal fat dynamics post-cavitation treatment. Employing a robust dataset with abdominal fat measurements and cavitation treatment parameters, we evaluate the efficacy of our approach through regression analysis. The performance of Hyperopt and Optuna regression models is assessed using metrics such as mean squared error, mean absolute error, and R-squared score. Our results reveal that both models exhibit strong predictive capabilities, with R-squared scores reaching 94.12% and 94.11% for post-treatment visceral fat, and 71.15% and 70.48% for post-treatment subcutaneous fat predictions, respectively. Additionally, we investigate feature selection techniques to pinpoint critical predictors within the fat prediction models. Techniques including F-value selection, mutual information, recursive feature elimination with logistic regression and random forests, variance thresholding, and feature importance evaluation are utilized. The analysis identifies key features such as BMI, waist circumference, and pretreatment fat levels as significant predictors of post-treatment fat outcomes. Our findings underscore the effectiveness of hyperparameter optimization in refining fat prediction models and offer valuable insights for the advancement of non-invasive fat reduction methods. This research holds important implications for both the scientific community and clinical practitioners, paving the way for improved treatment strategies in the realm of body contouring.

## Introduction

The pursuit of non-invasive techniques for body contouring and fat reduction has gained significant momentum in the medical and aesthetic fields. Among these techniques, cavitation—a process that utilizes ultrasonic waves to break down fat cells—has emerged as a promising method for altering abdominal fat composition^1,2^. Abdominal obesity, a key component of metabolic syndrome, poses significant health risks and is a critical factor in clinical diagnosis. The deposition of fat in the abdominal region, particularly visceral adipose tissue (VAT), is associated with an increased risk of obesity-related diseases and cardiometabolic disorders. VAT is known for its metabolic activity and pro-inflammatory properties, making it a prime target for therapeutic interventions^3–5^. Adipose tissue, primarily composed of adipocytes, serves essential functions such as energy storage in the form of lipids and thermal insulation. The distribution and volume of adipose tissue are influenced by a complex interplay of physiological, psychological, and clinical factors^6–8^. Two distinct types of adipose tissue—subcutaneous adipose tissue (SAT) and VAT—differ anatomically and functionally within the human body^9,10^. Cavitation therapy, utilizing low-frequency, high-energy ultrasound (US), has emerged as a promising non-invasive technique for reducing abdominal fat and enhancing body contouring. This approach targets adipose tissue, causing adipocyte disruption and subsequent release of triglycerides, which are then metabolized and excreted naturally. The efficacy of cavitation is contingent upon optimizing treatment parameters such as frequency, intensity, and duration, as well as considering the unique properties of adipose tissue^11,12^. To evaluate the impact of cavitation on abdominal fat, researchers utilize various medical imaging techniques, including ultrasound, CT, MRI, and DXA. These methods provide detailed assessments of fat distribution and volume, facilitating the monitoring of treatment outcomes^13^. The optimization of fat prediction models stands as a central focus in this research, employing advanced hyperparameter optimization techniques. Hyperopt and Optuna are used to refine these prediction models. The objective is to enhance the predictive accuracy of abdominal fat dynamics following cavitation treatment. By utilizing a robust dataset comprising measurements of abdominal fat and parameters of cavitation treatment, a regression analysis is conducted to evaluate the efficacy of the approach. Furthermore, various feature selection techniques are explored to identify the most critical predictors within the fat prediction models. This study investigates the application of cavitation in non-invasive abdominal fat reduction and body contouring, aiming to harness the power of machine learning to predict changes in abdominal fat post-treatment. A variety of techniques are explored, including F-value selection, mutual information, recursive feature elimination with logistic regression and random forests, variance thresholding, and feature importance evaluation. The analysis identifies key features such as body mass index (BMI), waist circumference, and pretreatment fat levels as significant predictors of post-treatment fat outcomes. The findings underscore the effectiveness of hyperparameter optimization in refining fat prediction models and offer valuable insights for the advancement of non-invasive fat reduction methods. This research holds important implications for both the scientific community and clinical practitioners, paving the way for improved treatment strategies and personalized care in the realm of body contouring.

### Challenges in fat prediction

Predicting body fat composition is a complex task that presents several challenges. First and foremost, the heterogeneity of human bodies makes it difficult to create a one-size-fits-all prediction model. Individuals vary greatly in terms of genetics, lifestyle, diet, and exercise habits, all of which influence body fat percentage. Additionally, the accuracy of the data collected, such as caloric intake and physical activity, often relies on self-reporting, which can be prone to errors and biases. Another challenge is the dynamic nature of body composition, which can change rapidly in response to various factors, making it hard to predict long-term outcomes accurately. The integration of different types of data, from biochemical markers to imaging data, while enriching the analysis, also adds complexity to the modeling process. Machine learning models require large amounts of high-quality, diverse data to be trained effectively, and such datasets are often difficult to obtain due to privacy concerns and the cost of comprehensive data collection. Moreover, the selection of appropriate machine learning algorithms that can handle the non-linearity and high dimensionality of the data without overfitting is a significant challenge. Ensuring that the models are interpretable and can provide actionable insights to healthcare providers is also crucial. Lastly, ethical considerations and the potential for algorithmic bias must be addressed to prevent the perpetuation of inequalities in healthcare outcomes. These challenges highlight the need for a multidisciplinary approach to develop robust, accurate, and fair models for fat prediction. This study delves into the utilization of machine learning algorithms for predicting obesity, with a focus on enhancing early detection and risk assessment.

### Problem statement and research gap

While there have been significant strides in the development of predictive models for obesity, a notable research gap exists in the precision and personalization of these models' predictions regarding treatment effects. Existing models offer a generalized approach but fall short of capturing the diverse responses individuals may have to obesity interventions. This highlights the necessity for more advanced models that can accommodate the unique characteristics of each patient's profile. The identified research gap lies in the current models' limited ability to deliver precise and tailored forecasts for obesity risk and the efficacy of treatment options. To bridge this gap, there is a pressing need for the creation of models that can assimilate a broader spectrum of data specific to the individual and apply sophisticated machine-learning techniques to significantly improve the accuracy of predictions.

### Research question

How can machine learning algorithms be optimized to improve the prediction of obesity and the individualized estimation of treatment effects?

### Contributions and research outline

The main contributions of this study can be summarized as:

- Developing machine learning models that provide more accurate predictions of obesity risk.
- Proposing a framework for individualized treatment effect estimation.
- Demonstrating the application of hyperparameter optimization techniques to improve model performance.

The organization of the paper is as follows: Section "Related work" encompasses a detailed examination of the literature pertinent to the study's domain. In Section "Materials and proposed methods", the Materials and Proposed Methods are thoroughly described, including the design of the clinical trial, the methodology for calculating the sample size, the strategies for recruiting participants, the selection of outcome measures, the regression techniques applied, and the dataset's features, along with the proposed work. Section "Experimental results and discussion" is devoted to the presentation of the Experimental results and includes an analysis and discussion of these findings. Section “Limitations” delves into the study's limitations. The paper culminates with Section “Conclusions and future directions”, summarizing the main discoveries and their implications for the advancement of personalized healthcare and the prediction of obesity treatment outcomes, and it also proposes potential avenues for future research.

## Related work

Prediction of possible outcomes and prediction of treatment impact are two important components of today's medical care and customized healthcare; forecasts of the absolute risk of a future occurrence under a variety of circumstances provide a natural basis for shared decision-making. The effect of treatment may be different for every individual, and thus it may be advantageous to customize or individualize its estimation. Machine learning aids in the discovery of better explanations for data and the prediction of the future based on previously acquired data. This section is a summary of prior related articles on obesity prediction.

Table 1 presents a summary of selected articles that explore the application of machine learning algorithms in predicting obesity and overweight. These studies employ various algorithms and feature sets to develop predictive models, aiming to improve early identification and risk assessment of these conditions. The accuracy of these models is reported, providing insights into the performance and potential utility of machine learning in addressing the obesity epidemic.

**Table 1:** A summary of machine learning algorithms used in predicting obesity and overweight.

Authors	Year	Summary	Algorithm	Dataset size	Accuracy/result
Liu et al.^14^	2013	This study investigated the distribution of fat in the trunk and established regression equations to estimate visceral fat (VF) and subcutaneous fat (SF)	SPSS17.0 to conduct multiple regression analysis	51	The study identified gender-specific optimal locations (2 cm above L4-L5 for men, 7–8 cm above L4-L5 for women) to measure VF for better fat distribution assessment
Chen et al.^15^	2014	This study aimed to develop a quick and accurate method for estimating visceral fat area (VFA) in the L4-L5 vertebrae using easily measurable anthropometric variables	Correlation analysis	227	92%
Dugan et al.^16^	2015	This study investigated the use of machine learning to predict childhood obesity after the age of two, using data collected before the second birthday through a clinical decision support system (CHICA)	Decision tree (ID3)	7519	85%
Sun et al.^17^	2017	This study investigated the use of novel 3D body shape descriptors to predict abdominal fat (visceral and subcutaneous adipose tissue)	Multiple regression analysis	121	The final prediction equations explained 74.2% of the variance of VAT in men and 80.4% in women
Rina So et al.^18^	2017	This study aimed to develop a new equation for estimating abdominal visceral adipose tissue (VAT) volume using readily available anthropometric measurements collected during workplace health checkups. Additionally, it investigated the association between VAT volume and metabolic risk factors	Multiple regression analysis	260	Demonstrated a moderate correlation (r = 0.74) between measured and predicted VAT volumes, suggesting the potential effectiveness of the equation
Montanez et al.^19^	2017	This study investigated the use of machine learning and genetic data to predict obesity risk based on body mass index (BMI) and presented an approach that combines genetic data analysis with machine learning for obesity risk prediction	SVM	6622	90.5%
Zheng et al.^20^	2017	This study used machine learning to predict obesity in high school students based on both risk and protective health behaviors	Logistic regression, Decision tree, weighted K-Nearest Neighbor, and artificial neural network	5227	88.82%
Jindal et al.^21^	2018	This study investigated the use of an ensemble machine learning model to predict obesity levels based on various factors	Ensemble machine learning	600	89.68%
Taghiyev et al.^22^	2020	This study focused on developing a more accurate method to identify factors contributing to obesity in females in the Aksaray Sultanhani region of Turkey	Decision trees (DT) and Logistic regression (LR)	500	91.4%
Rodriguez et al.^23^	2021	This study investigated the use of machine learning to develop a model for identifying people with overweight or obesity	Random forest	2111	78%
Kivrak^24^	2021	This study investigated the use of deep learning methods to predict obesity levels from a publicly available dataset of patient records	CNN	17 variables	82%
Proposed work	2024	The study highlights the effectiveness of hyperparameter optimization in improving fat prediction models for non-invasive fat reduction. It pinpoints key factors affecting fat reduction and paves the way for better body contouring treatments. These findings are valuable for both researchers and medical professionals	Hyperopt and Optuna	63	Post-treatment visceral fat: R-squared scores of 94.12% (Hyperopt) and 94.11% (Optuna) Post-treatment subcutaneous fat: R-squared scores of 71.15% (Hyperopt) and 70.48% (Optuna)

## Materials and proposed methods

### Trial design

The current research was intended to be a clinical trial. This research was authorized by the Deraya University Ethical Committee (No: 17/2023). According to the Helsinki Declaration's ethical standards. This study adheres to human research principles. Following a full explanation of the trial, all participants signed a written consent form. From February 2023 until July 30th, 2023, the trial was held at a medical clinic for outpatients.

### The sample size

To prevent type II error, a sample size calculation was performed before the study using the G*Power (Wilcoxon–Mann–Whitney test)^25^. This output displays the results of an a priori power analysis for a linear regression t-test. The purpose is to determine the minimum required sample size needed to achieve a desired statistical power of 0.95 (95%).

Specifically:

- A one-tailed t-test is specified to detect an alternative slope (H1) of 0.1732051 as greater than the null slope of 0.
- An alpha error probability of 0.05 is set.
- The desired power is 0.95, with standard deviations and null/alternative slopes provided.

The analysis calculates a no centrality parameter δ of 3.3665016 based on these inputs.

It then determines the critical t-value of 1.6938887 and degrees of freedom of 32 needed to achieve ≥ 0.95 power. The total required minimum sample size to meet these conditions is calculated as 34 observations. The actual estimated power computed from these parameters is 0.9504455, exceeding the target of 0.95 power. Therefore, this output provides the minimum sample size (N = 34) required to have a 95% chance of correctly detecting a statistically significant slope of 0.1732051 in a one-tailed linear regression t-test at the 5% significance level. This means that the study has enough power to identify a significant difference between the two measures with high confidence. As a result, the study has an acceptable sample size, and the results are reliable and genuine.

### Participants

The sixty-three participants in the research study were initially diagnosed with abdominal obesity and recruited from the clinical nutrition department of the General Hospital. Recruitment was based on the following criteria: females and males participated in this trial; their ages varied from 25 to 45 years, their BMI was 25–29.9 kg/m^2^, and the participants were not treated with lipolytic drug therapy.

### Exclusion criteria

Any prior medical history of cardiopulmonary disease, disc prolapse, disease of the liver or kidneys, gastric or gallbladder ulcer, diabetes mellitus, cigarette smoking, cognitive impairments, patients who have peacemaker or any type of metal implant on the treated area, cancer or patients with a history of tumor and any surgery related to the spine, abdomen, or pelvis.

### Outcome measures

All of the participants were evaluated before and after two months of intervention, with two sessions each week. Anthropometry is the measurement of one’s weight, height, waist circumference, and calculated BMI***.***

#### Waist circumference

Non-elastic, tight, and 150-cm tape is used. The distance was measured midway between the base of the lower rib and the top of the iliac crest. Waist circumference is an indicator of central obesity, which is where adipose tissue is deposited^4^.

#### Body mass index

The body mass index (BMI) for all participants was calculated using the following equation using a universal height-weight scale to ascertain the subject’s height and weight. BMI (kg/m^2^): weight (kg)/heigh2 (m^2^), a global classification of BMI values based on a set of cut-off criteria for weight conditions: 18.5 lbs underweight; 18.5–25.0 lbs normal weight; 25.0 lbs overweight^5^.

#### Ultrasonography examination for subcutaneous fat

The various acoustic characteristics of different tissues are used in ultrasound imaging. The patient was lying supine for the measurement. At the beginning of the examination, any air bubbles were removed by soaking the probe tip in saline and gently massaging the tip with a bent swab. To eliminate obliquity and inaccuracies during skin thickness measurement, the transducer was placed perpendicular to the skin during imaging. A thick layer of US gel is applied to increase near-field visibility and reduce tissue compression, which will change tissue thickness measurements. The sonographer performs an ultrasonographic examination on all individuals twice, before and after each session of treatment^28^. The image was obtained in the abdominal area para umbilical region 2 cm lateral to umbilicus while the participant stopped breathing during mid-tidal expiration. The epidermis-muscle tissue distance was measured. The examiner evaluated each of the measurements on two planes: the first parallel to the longitudinal axis of the abdomen, and the second perpendicular to the first^29^.

#### Ultrasonography examination for visceral fat

The patient was positioned supine for ultrasound imaging, and a thick layer of gel was placed on the probe. The probe was placed 2 cm above the umbilicus in the transverse plane. The distance was determined by measuring from the lower border of the abdominal muscle to the higher border of the pulsing aorta^30^.

### Treatment procedures

Using an ultrasonic cavitation machine (Cavi –SMART, South Korea), supplied with specific parameters, Frequency: 40 kHz, Ultrasonic output power: 50W, Ultrasonic Output mode: hand-held treatment head (50 mm diameter, round stainless Steel), Size: 450 × 300 × 250 mm and weight: 7 kg. After taking a comprehensive history from each participant, a follow-up assessment and recording of the parameters for each subject were conducted at the beginning and end of the study period (two months). Anthropometric measurements: Patients’ height and weight were measured while wearing a light layer of clothing and bare feet. Body mass index was calculated by dividing the weight in kilograms by the measure of the patient’s height in meters, and waist circumference was measured while standing in an erect standing position with feet together.

A preliminary visit is performed to identify adipose tissue with a thickness of at least 2 cm; the patients are provided with information about the treatment, and medical screening is performed to ensure that the patients do not have any problems caused by dyslipidemic or hepatic diseases, tumoral and autoimmunity disorders, or skin diseases in the areas to be treated. Each woman was asked to clear her bladder on the session day before beginning treatment to ensure that she was able to remain calm. The patient is comfortably positioned on a bed after the area to treat has been signed with appropriate demographics pencils, avoiding lowering the thickness of the Adipose tissue that develops as a result of elevated skin tensions caused by potential underlying bone prominence. The patients were then placed in a supine position for the session. The treatment area is then isolated with small surgery sheets and covered with conductive gel to help the ultrasound waves spread; it also acts as a coupling mean probing skin, avoiding reflection problems^31^. To get the desired effect, localized fat was treated twice per week for two continuous months—the treatment area was the abdomen area with an average time of 20–30 min for each area per session.

### Methodology

#### Regression techniques

When performing regression analysis, it’s crucial to select the appropriate model and techniques to achieve accurate predictions. In this introduction, we will provide an overview of several regression models along with their descriptions, the steps involved, and the pros and cons associated with each approach. Table 2 summarizes the regression techniques used in the study.

**Table 2:** Summary of the regression techniques used in the study.

Model	Description	Steps	Pros	Cons
Hyperopt regression^32^	Regression with hyperopt	Hyperparameter tuning	Automatic hyperparameter tuning Can improve model performance Provides a variety of search spaces Supports parallel computing	Computationally expensive May lead to overfitting if not careful Requires careful interpretation of results Limited by the search space definition
Optuna regression^33^	Regression with optuna	Hyperparameter tuning	Efficient hyperparameter search User-friendly interface Visualization of the tuning process Supports pruning of unpromising trials	Limited to optimization Can be time-consuming Requires multiple runs for best results Pruning may discard potentially good candidates
Hybrid regression^34^	Regression with hybrid metrics	Combination of multiple metrics	Improved accuracy through metric combination Flexible model design Can capture complex patterns Adaptable to various data types	Complexity in metric selection Risk of model overfitting May require extensive computational resources Potentially high model complexity
ElasticNetCV^35^	Elastic Net regression	Cross-validation	Handles multicollinearity Combines L1 and L2 regularization Can select relevant features Robust to outliers	Requires optimization Sensitive to scale of data Can be outperformed by non-linear models Selection of hyperparameters is crucial
RandomForest regressor^36^	Random Forest regression	Ensemble learning	Nonlinear relationships Robust to outliers Handles high-dimensional data well Provides feature importance scores	May overfit Model size can become large Computationally intensive Performance can decrease with noisy data
SVR^37^	Support Vector Regression	Feature scaling	Effective for high-dimensional data Robust to overfitting Can handle non-linear relationships Provides sparse solutions	Sensitive to hyperparameters Requires feature scaling Long training time for large datasets Difficult to interpret model results
BAGGINGREGRESSOR^38^	Bagging regression	Ensemble learning	Reduces variance Can improve accuracy Robust to noise and outliers Easy to parallelize	May have a high computational cost Model predictions can be complex Requires careful tuning Not as interpretable as simpler models
K Neighbors regressor^39^	k-Nearest Neighbors regression	Feature scaling	Captures local patterns Simple to implement No assumptions about data distribution Effective if the number of features is small	Computationally intensive for large datasets Sensitive to irrelevant features Requires feature scaling Performance degrades with dimensionality increase

#### Dataset characteristics

The given dataset provides measurements for various parameters related to individuals, including sex, age, weight, height, BMI, waist circumference, pretreatment visceral fat, posttreatment visceral fat, pretreatment subcutaneous fat, and posttreatment subcutaneous fat. Let's describe each column in detail:

1. **Sex:** This column represents the biological sex of the individuals in the dataset. The letter "M" denotes male, and the letter "F" denotes female. It indicates the gender identity of each person.
2. **Age:** This column specifies the age of each individual in years. It provides information about the chronological age of the person at the time of measurement.
3. **Weight:** The weight column represents the measured weight of each person in kilograms. It indicates the mass or heaviness of the individual.
4. **Height:** The height column represents the measured height of each person in centimeters. It indicates the vertical stature or tallness of the individual.
5. **BMI:** BMI stands for Body Mass Index, and it is calculated by dividing the weight (in kilograms) by the square of height (in meters). The BMI column provides the calculated BMI value for each individual. It is a numerical measure that helps assess whether a person has a healthy weight, is underweight, overweight, or obese.
6. **Waist circumference:** This column represents the measurement of the waist circumference of each individual in centimeters. Waist circumference is used as an indicator of abdominal or central obesity.
7. **Pretreatment visceral fat:** Visceral fat refers to fat that is stored around internal organs in the abdominal cavity. The pretreatment visceral fat column provides the measurement of the amount of visceral fat (in arbitrary units) before a specific treatment or intervention.
8. **Posttreatment visceral fat:** Similar to the pretreatment visceral fat column, this column represents the measurement of the amount of visceral fat (in arbitrary units) after the treatment or intervention. It helps assess the effectiveness of the treatment in reducing visceral fat.
9. **Pretreatment subcutaneous fat:** Subcutaneous fat refers to the fat stored under the skin. The pretreatment subcutaneous fat column provides the measurement of the amount of subcutaneous fat (in arbitrary units) before the treatment or intervention.
10. **Posttreatment subcutaneous fat:** This column represents the measurement of the amount of subcutaneous fat (in arbitrary units) after the treatment or intervention. It helps assess the effectiveness of the treatment in reducing subcutaneous fat.

**Figure 1:**
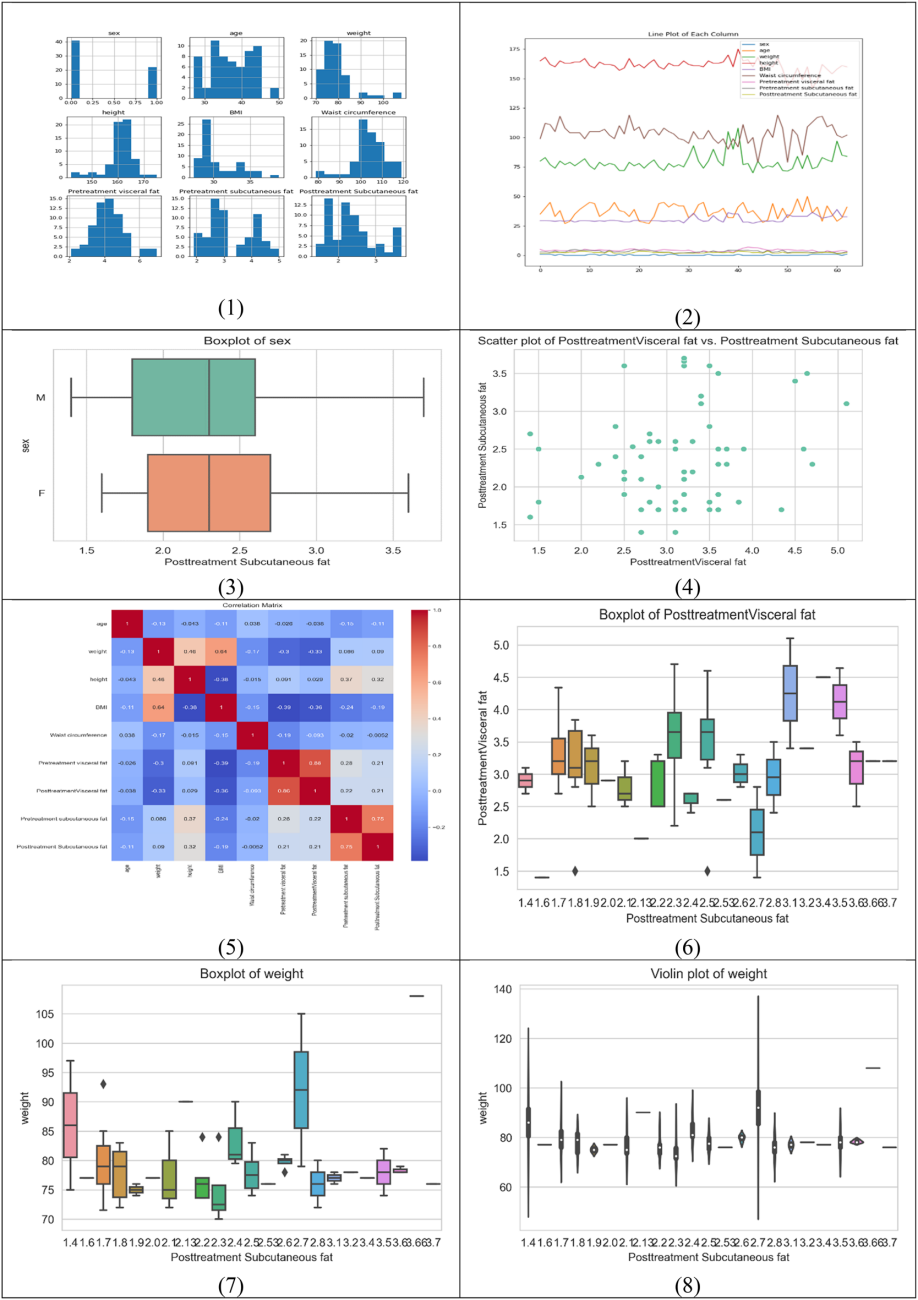
Correlation between dataset features.

Each row in the dataset corresponds to a specific individual, and the values in each column represent the respective measurements for that individual. The data includes 63 observations, each with 10 columns. Table 3 presents descriptive statistics for each feature in the dataset, which includes the number of features, mean, median, standard deviation, minimum, 25th percentile, 50th percentile (median), 75th percentile, and maximum values and Fig. 1 shows the correlation between the dataset features.

**Table 3:** Descriptive statistics of the dataset features.

Statistic	Age	Weight	Height	BMI	Waist circumference	Pretreatment visceral fat	Posttreatment visceral fat	Pretreatment subcutaneous fat
Mean	37.25	79.27	161.02	30.59	103.35	4.27	3.13	3.27
Median	37.00	78.00	161.00	29.38	104.00	4.30	3.20	2.90
Standard deviation	5.75	7.17	5.56	2.58	8.10	0.95	0.77	0.83
Minimum	27.00	70.00	142.00	27.30	79.00	2.11	1.40	1.90
25th percentile	33.00	75.00	159.50	28.95	99.00	3.70	2.70	2.70
50th percentile (median)	37.00	78.00	161.00	29.38	104.00	4.30	3.20	2.90
75th percentile	42.00	80.50	164.00	32.60	108.00	4.60	3.55	4.21
Maximum	50.00	108.00	175.00	38.90	119.00	7.00	5.10	5.00
Variance	33.10	51.37	30.92	6.67	65.57	0.90	0.38	0.69

Table 4 shows the relationship between the numerical variables in the dataset. Each row and column in the matrix represents a continuous variable, and Pearson's R-value corresponding to that row and column reflects the strength and direction of the correlation between the variables. Most qualities are significantly connected, according to our observations. This matrix provides an in-depth look at the correlations between various attributes, with each attribute listed on both the rows and columns. The numbers in the rows and columns show the correlation coefficient between the two traits, with a coefficient close to 1 representing a high positive correlation, a coefficient close to -1 representing a strong negative correlation, and a coefficient close to 0 representing no association.

**Table 4:** The correlation heat map of the proposed framework.

	Weight	Height	BMI	Waist circumference	Pre-treatment visceral fat	Post-treatment of visceral fat	Pre-treatment subcutaneous fat	Post-treatment subcutaneous fat
Weight	1.000	0.464	0.63	− 0.170	− 0.296	− 0.328	0.086	0.090
Height	0.464	1.000	− 0.383	− 0.015	0.091	0.029	0.369	0.316
BMI	0.639	− 0.38	1.00	− 0.151	− 0.387	− 0.358	− 0.241	− 0.193
Waist circumference	− 0.170	− 0.01	− 0.15	1.000	− 0.194	− 0.093	− 0.020	− 0.005
Pretreatment visceral fat	− 0.296	0.091	− 0.38	− 0.194	1.000	0.864	0.275	0.212
Post treatment visceral fat	− 0.328	0.029	− 0.35	− 0.093	0.864	1.000	0.218	0.214
Pretreatment subcutaneous fat	0.086	0.369	− 0.24	− 0.020	0.275	0.218	1.000	0.754
Post treatment subcutaneous fat	0.090	0.316	− 0.19	− 0.005	0.212	0.214	0.754	1.000
BMI	0.639	− 0.38	1.00	− 0.151	− 0.387	− 0.358	− 0.241	− 0.193

#### The proposed framework

**Figure 2:**
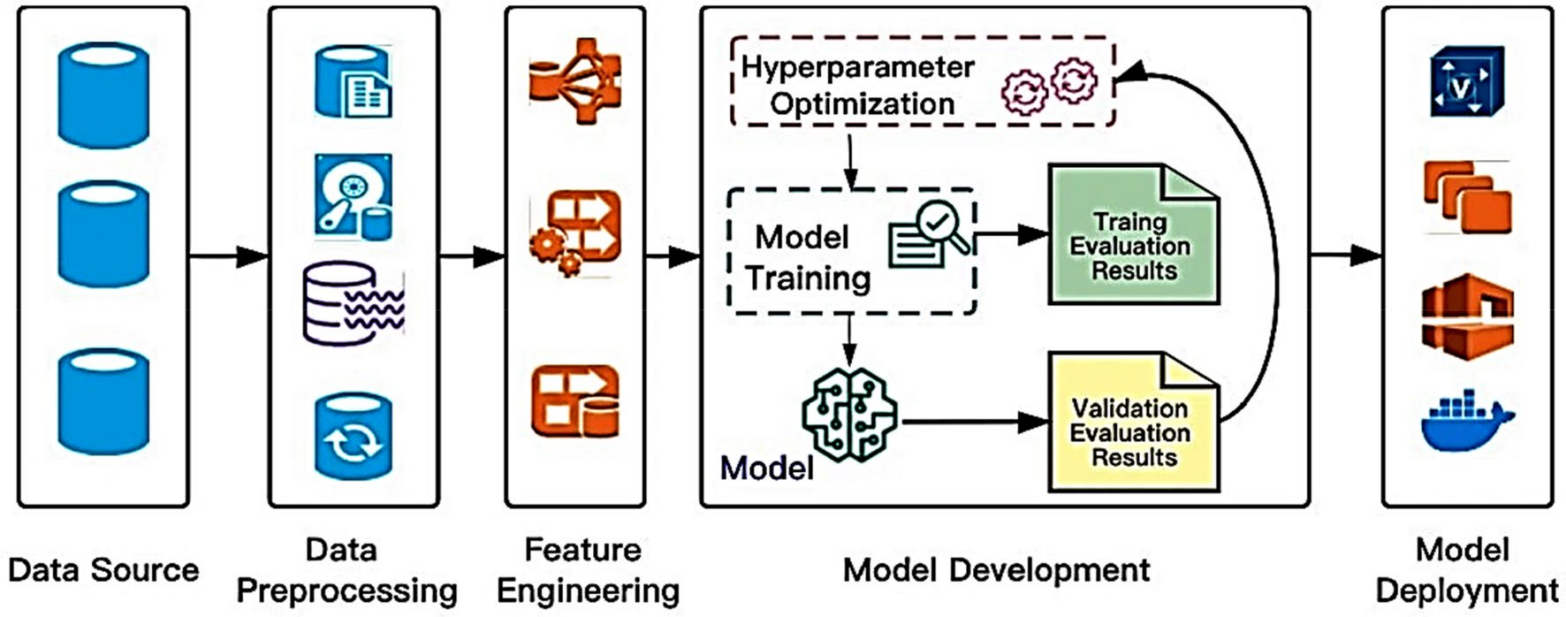
A high-level depiction of a typical machine learning workflow and the role of hyperparameter optimization algorithms^40^.

Hyperparameter optimization algorithms are pivotal in boosting the performance of machine learning models. The workflow typically encompasses several stages, starting with the collection and preprocessing of raw data from diverse sources. Following this, feature engineering is conducted to ensure the derived features are conducive to efficiently training machine learning models. In the initial phase, it is prudent to opt for a straightforward yet effective technique to train the initial baseline model during the maiden iteration. Evaluating the baseline model against predefined accuracy and business value metrics elucidates its comparative performance^40^. Once deployed, continuous monitoring of the model's performance in the production environment enables iterative enhancements in subsequent iterations as shown in Fig. 2.

**Figure 3:**
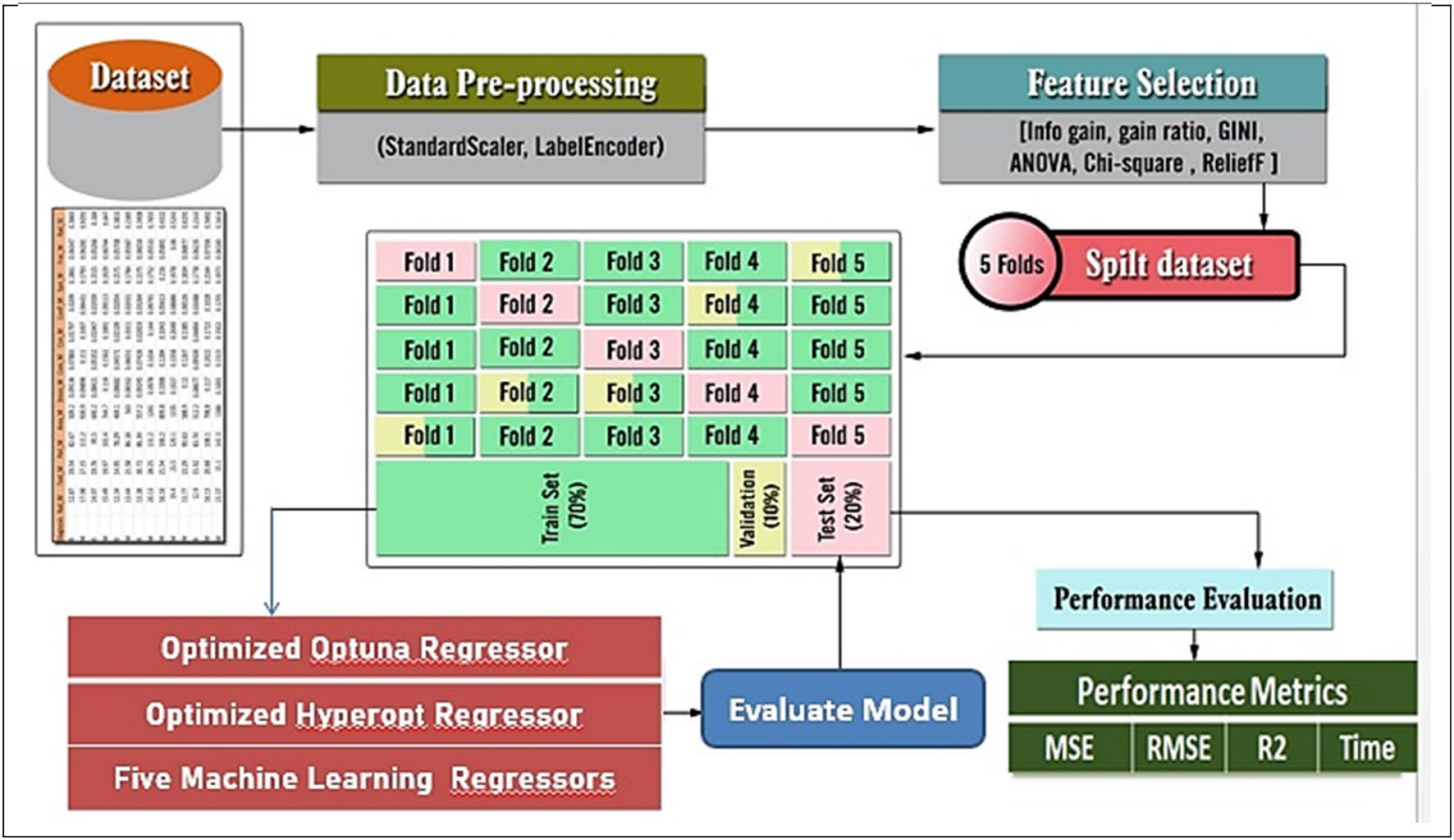
The general framework of the proposed prediction model.

**Figure 4:**
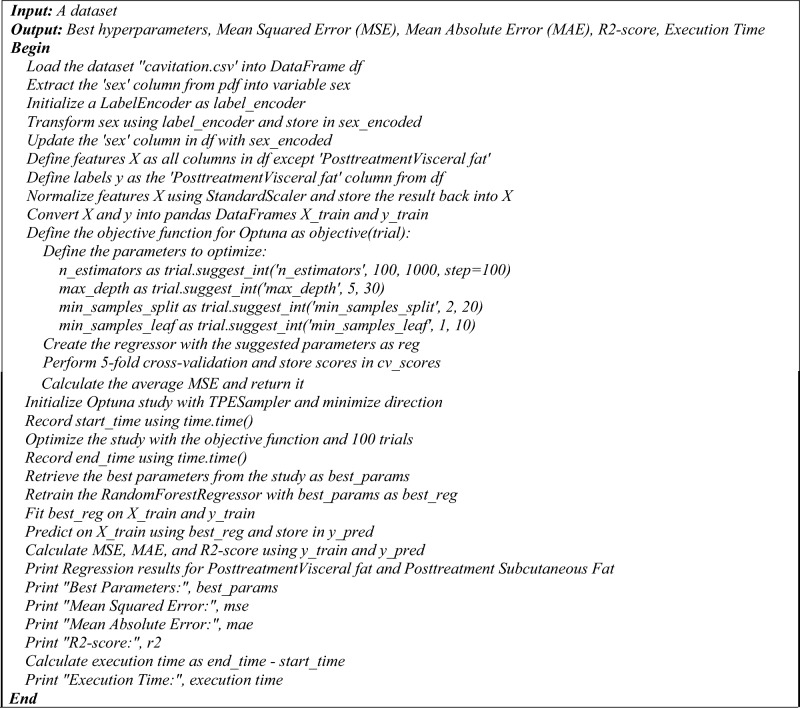
The pseudocode of the proposed hyperopt regression model.

**Figure 5:**
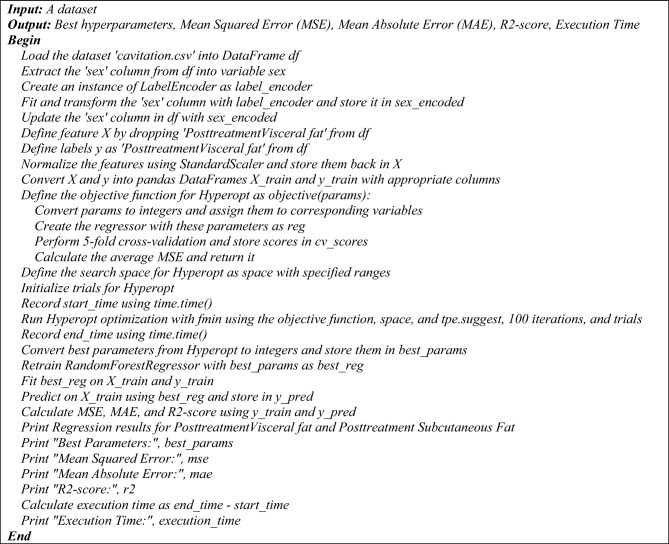
The pseudocode of the proposed hyperopt regression model.

Figure 3 illustrates the proposed framework's structure, which includes the prediction process as well as the performance metrics. Figures 4 and 5 show pseudocode representations of the proposed Optuna and Hyperopt optimizers. The diagram depicts a typical machine learning workflow, where data is collected, and pre-processed, features are selected, the data is split, and various machine learning models are trained and evaluated on the data. The components of Fig. 3 can be summarized as follows:

- **Dataset:** This is the initial collection of data that the machine learning system will be trained on. It is depicted as a rectangular box at the top of the image, labeled "Dataset".
- **Data pre-processing:** This stage involves cleaning and preparing the data for use in the machine learning model. It is shown as a rectangular box with a dashed line around it, branching off to the right from the "Dataset" box. It includes two methods: "StandardScaler" and "LabelEncoder".
- **Feature selection:** This stage involves selecting the most relevant features from the data to be used in the model. It is depicted as a rectangular box with a dashed line around it, branching off to the right from the "Data Pre-processing" box. It shows multiple methods including "Info gain", "gain ratio", "GINI", "ANOVA", "Chi-square", and "ReliefF".
- **Splitting the data:** This stage involves dividing the data into different sets for training, validation, and testing the machine learning model. It is shown as a section below the "Data Pre-processing" box, where the data splits into five sections labeled "Fold 1" through "Fold 5".
- **Machine learning algorithms:** These are the algorithms that will be used to learn from the data and make predictions. They are depicted as a rectangular box at the bottom left of the image, labeled "Five Machine Learning Regressors".
- **Performance evaluation:** This stage involves evaluating the performance of the machine learning models on the test data. It is shown as a rectangular box at the bottom right of the image, labeled "Performance Evaluation". It includes metrics like "MSE", "RMSE", "R2", and "Time".

The pseudocode provided outlines two distinct regression processes utilizing a Random Forest Regressor with two different hyperparameter tuning methods: Optuna and Hyperopt. Both algorithms aim to optimize the hyperparameters of the regression model to predict post-treatment visceral fat based on a dataset, and they output the best hyperparameters along with performance metrics such as MSE, MAE, R2-score, and execution time.

**Optuna hyperparameter optimization for Random Forest regression**

The process begins by loading the dataset 'cavitation.csv' into a DataFrame and preparing the data. The 'sex' column is extracted, encoded using a LabelEncoder, and updated back into the DataFrame. Features and labels are defined, with features being normalized and both features and labels converted into training DataFrames. An objective function is defined for Optuna, which specifies the hyperparameters to be optimized, such as 'n_estimators', 'max_depth', 'min_samples_split', and 'min_samples_leaf'. A Random Forest Regressor is created with these parameters, and fivefold cross-validation is performed to calculate the average MSE. An Optuna study is initialized and optimized using the objective function over 100 trials. The best parameters are retrieved, and the RandomForestRegressor is retrained and fitted on the training data. Predictions are made, and performance metrics are calculated and printed, along with the execution time.

**Hyperopt hyperparameter optimization for Random Forest regression**

Similarly, the Hyperopt process starts by loading the dataset and preparing the data in the same manner as the Optuna process. The objective function for Hyperopt is defined to convert parameters, create the regressor, perform cross-validation, and calculate the average MSE. The search space for Hyperopt is defined with specified ranges for the hyperparameters. Hyperopt optimization is run using the 'fmin' function with the objective function, search space, 'tpe. suggest', and 100 iterations. The best parameters from Hyperopt are retrieved, and the Random Forest Regressor is retrained and fitted on the training data. Predictions are made, and performance metrics are calculated and printed, along with the execution time.

Both pseudocodes conclude with the printing of regression results for post-treatment visceral and subcutaneous fat, best hyperparameters, MSE, MAE, R2-score, and the execution time. These pseudocodes serve as a step-by-step guide for implementing the proposed regression models with hyperparameter optimization. The following is a pseudocode of the proposed regression process that uses a Random Forest Regressor and Hyperopt for hyperparameter tuning:

#### Evaluation metrics for regression and classification models

**Evaluation metrics for regression models**: The determination coefficient R-square is one of the most common performances used to evaluate the regression model as shown in Eq. (1). On the other hand, the Minimum Acceptable Error (MAE) is shown in Eq. (2), while the Mean Square Error (MSE) is investigated in Eq. (3).

1 $${{\text{R}}}^{2}=\frac{\sum {\left(y-\dot{\widehat{y}}\right)}^{2}}{\sum {\left(y-\dot{\overline{y}}\right)}^{2}}$$

2 $${\text{MAE}}=\frac{\sum_{i=1}^{n}\left|\widehat{{y}_{i}}-y\right|}{{\text{n}}}$$

3 $${\text{MSE}}=\frac{\sum_{i=1}^{n}{({y}_{i}-\widehat{{y}_{i}})}^{2}}{{\text{n}}}$$

where y is the actual value, $\dot{\widehat{{\text{y}}}}$ is the corresponding predicted value, $\dot{\overline{{\text{y}}}}$ is the mean of the actual values in the set, and ***n*** is the total number of test objects^41–43^.

**Index of Agreement:** Willmott proposed an index of agreement (d) as a standardized measure of the degree of model prediction error which varies between 0 and 1. The index of agreement represents the ratio of the mean square error and the potential error. The agreement value of 1 indicates a perfect match, and 0 indicates no agreement at all. The index of agreement can detect additive and proportional differences in the observed and simulated means and variances; however, d is overly sensitive to extreme values due to the squared differences as shown in Eq. (4)^44^.

4 $$d=1-\frac{\sum_{i=1}^{n}{\left({O}_{i}-{P}_{i}\right)}^{2}}{\sum_{i=1}^{n}{\left({|{P}_{i}-\overline{O} }_{i}\left|+{|{O}_{i}-\overline{O} }_{i}\right|\right)}^{2}}, 0\le d\le 1$$

where O_i_ is the observation value P_i_ is the forecast value O bar is the average observation value and P bar is the average forecast value.

**Statistical analysis: Posthoc Nemenyi test:**

The post hoc Nemenyi test is a multiple comparison test that allows us to compare the pairs of models to determine which pairs are significantly different. The test produces a test statistic called the Nemenyi statistic, which is calculated as in Eq. (5).

5 $$Nermenyi statistic= {\left(\frac{a}{b}\right)}^{2}{-\left(\frac{c}{d}\right)}^{2}$$

where a and b are the accuracies of two models being compared, and c and d are the times required to achieve those accuracies. The p-value for the Nemenyi test is calculated as in Eq. (6).

6 $$p-value = P(Nemenyi \mathrm{statistic }>\mathrm{ observed Nemenyi statistic}) $$

To determine the best model using statistical analysis, you can perform a post hoc Nemenyi test. The Nemenyi test is a non-parametric statistical test used for multiple comparisons of mean ranks. It can be used to determine if there are significant differences between the models based on the performance measures.

The step-by-step process to perform the posthoc Nemenyi test:

Step 1: Rank the models based on their performance measures. In this case, we can use the mean squared error (MSE), mean absolute error (MAE), and R-squared score.

Step 2: Calculate the average rank for each model across the three performance measures.

Step 3: Calculate the critical difference (CD) value. The CD value represents the minimum difference between the average ranks that is considered significant. It depends on the number of models and the significance level chosen.

Step 4: Compare the average ranks of the models pairwise and check if the difference is greater than the CD value. If the difference is greater, it indicates a significant difference between the models.

Step 5: Based on the results of the pairwise comparisons, identify the best model.

### Ethical statement

All procedures performed in studies involving human participants were by the ethical standards of the institutional and/or national research committee and with the 1964 Helsinki Declaration and its later amendments or comparable ethical standards. The study was authorized by the Deraya University Ethical Committee (No: 17/2023). Following a full explanation of the trial, each patient filed a written consent form. The research was carried out at the outpatient clinic from February 2023 to July 30, 2023.

### Consent statement

Informed consent was obtained from all individual participants included in the study.

## Experimental results and discussion

To evaluate the effectiveness of our machine learning framework, we conducted experiments in this section. The experiments were performed on a computer with a 3 GHz i5 processor, 8 GB main memory, and a 64-bit Windows 10 operating system. We used the Python programming language to experiment.

### The results of the proposed regression machine learning technique

**Figure 6:**
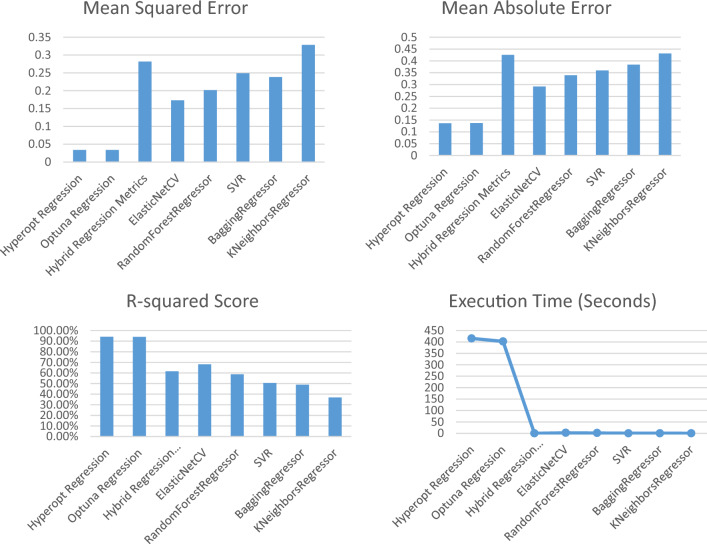
The performance metrics of the regression models of posttreatment visceral fat.

**Figure 7:**
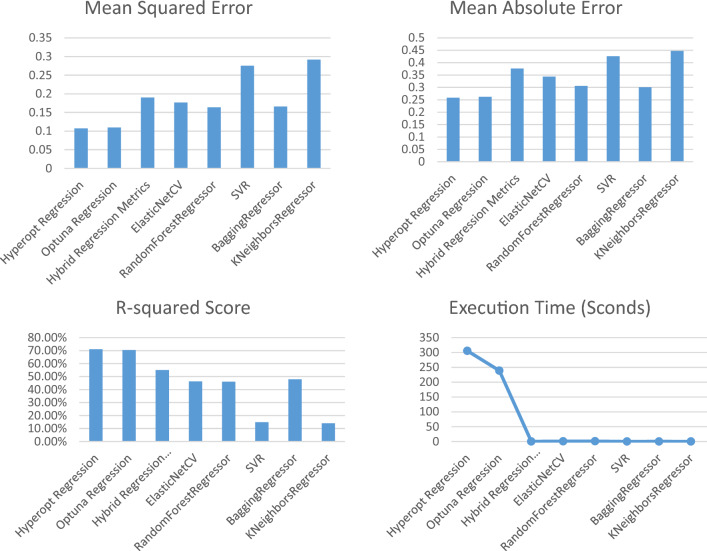
The performance metrics of the regression models of posttreatment subcutaneous fat.

Tables 5, 6, and Figs. 6, and 7 present the results of a regression task for Posttreatment Visceral Fat and Posttreatment Subcutaneous Fat using different machine learning models. The analysis and expansion of the Table can be summarized as follows:

- Model: this column shows the names of the machine learning models used in the regression task.
- MSE (mean squared error): this column represents the average of the squared differences between the predicted and actual values. A lower value of MSE indicates better performance.
- MAE (mean absolute error): this column represents the average of the absolute differences between the predicted and actual values. A lower value of MAE indicates better performance.
- R2 score: this column represents the coefficient of determination, which measures the proportion of variance in the target variable that can be explained by the independent variables. A higher value of the R2 Score indicates better performance.
- Index of agreement: this metric assesses the extent of prediction error generated by the model, ranging from 0 to 1. It is calculated as the ratio of the mean squared error to the potential error. A value of 1 signifies a perfect alignment between predictions and actual observations, while 0 indicates complete disagreement.
- Time taken (s): this column represents the amount of time taken by each model to complete the regression task.

**Table 5:** The evaluation of different regression models for Posttreatment Visceral Fat to assess their performance.

No	Model	Mean squared error	Mean absolute error	R-squared score (%)	Index of agreement	Execution time
1	Hyperopt regression	0.0341	0.1364	94.12	0.8925	415.62
2	Optuna regression	0.0341	0.1370	94.11	0.8919	402.37
3	Hybrid regression	0.2818	0.4254	61.56	0.4257	0.20
4	Elastic NetCV	0.1733	0.2918	68.11	0.6783	2.30
5	Random forest regressor	0.2019	0.3392	58.76	0.6028	1.67
6	SVR	0.2491	0.3594	50.52	0.4952	0.23
7	Bagging regressor	0.2385	0.3838	48.89	0.5111	0.22
8	K Neighbors regressor	0.3287	0.4312	36.89	0.3775	0.15

**Table 6:** The evaluation of different regression models for Posttreatment Subcutaneous Fat to assess their performance.

No	Model	Mean squared error	Mean absolute error	R-squared score (%)	Index of agreement	Execution time (s)
1	Hyperopt regression	0.1072	0.2585	71.15	0.7243	305.41
2	Optuna regression	0.1097	0.2623	70.48	0.7228	238.77
3	Hybrid regression	0.1899	0.3765	55.11	0.5583	0.19
4	ElasticNetCV	0.1768	0.3435	46.29	0.5087	0.89
5	Random Forest regressor	0.1637	0.3063	46.10	0.5282	1.03
6	SVR	0.2754	0.4261	14.84	0.3201	0.08
7	Bagging regressor	0.1658	0.3010	47.87	0.5297	0.18
8	K Neighbors regressor	0.2919	0.4475	14.04	0.3092	0.06

As shown in Table 5 and Fig. 6:

- Hyperopt Regression and Optuna Regression performed the best with the lowest MSE and MAE, and highest R-squared scores of 94.12% and 94.11% respectively. This demonstrates that these hyperparameter optimization techniques were able to find optimal hyperparameters for the regression model to best fit the data.
- Hybrid Regression had a higher MSE and MAE and lower R-squared score compared to Hyperopt/Optuna Regression, indicating it did not fit the data as well.
- ElasticNetCV, RandomForestRegressor, and SVR had moderately high R-squared scores between 58 and 68%, showing they could also predict the data reasonably well, though not as good as Hyperopt/Optuna.
- BaggingRegressor and KNeighborsRegressor had the lowest R-squared scores between 36.89 and 48.89%, suggesting they were not able to capture the relationships in the data as effectively as the other models.
- In terms of execution time, Hyperopt/Optuna Regression took the longest at over 400 s likely due to the intensive hyperparameter search. The other models were much faster, with KNeighborsRegressor being the quickest at under 0.2 s.

**Statistical analysis: posthoc Nemenyi Test for posttreatment visceral fat:**

The mean ranks for the given models based on the performance metrics:

- Hyperopt regression: mean rank = 1.25
- Optuna regression: mean rank = 2
- Hybrid regression: mean rank = 5
- ElasticNetCV: mean rank = 4.5
- Random forest regressor: mean Rank = 4.5
- SVR: mean rank = 7
- Bagging regressor: mean rank = 4.5
- KNeighbors regressor: mean rank = 7

To calculate the CD value, we need to determine the critical value for the Studentized range statistic. For the Nemenyi test with 8 models and 4 performance metrics, the critical value for a significance level of 0.05 is approximately 2.998.

Now, we can compare the mean ranks of each pair of models and check if the difference is greater than the CD value. Based on the comparisons, we can determine if there are significant differences between the models.

Comparisons:

- Hyperopt regression vs. Optuna regression: mean rank difference = 0.75 (greater than CD)
- Hyperopt regression vs. hybrid regression: mean rank difference = 3.75 (greater than CD)
- Hyperopt regression vs. ElasticNetCV: mean rank difference = 3.25 (greater than CD)
- Hyperopt regression vs. random forest regressor: mean rank difference = 3.25 (greater than CD)
- Hyperopt regression vs. SVR: mean rank difference = 5.75 (greater than CD)
- Hyperopt regression vs. bagging regressor: mean rank difference = 3.25 (greater than CD)
- Hyperopt regression vs. K neighbors regressor: mean rank difference = 5.75 (greater than CD)

Based on these comparisons, we can conclude that the Hyperopt Regression model is significantly different from all other models. There are significant differences between the Hyperopt Regression model and the Optuna Regression, Hybrid Regression, ElasticNetCV, RandomForestRegressor, SVR, BaggingRegressor, and KNeighborsRegressor models.

Therefore, based on the statistical analysis using the post hoc Nemenyi test, the Hyperopt Regression model can be considered the best model among the given options.

Hyperopt Regression and Optuna Regression achieved the best predictive performance on this dataset, demonstrating the benefit of leveraging advanced hyperparameter optimization techniques. Their long runtime was justified by the improved accuracy over faster but less optimized models.

As shown in Table 6 and Fig. 7:

- Hyperopt Regression and Optuna Regression again had the best metrics with the lowest MSE and MAE, and highest R-squared scores of 71.15% and 70.48% respectively.
- Their R-squared scores for subcutaneous fat prediction were lower than for visceral fat (94% +), indicating subcutaneous fat is more difficult to predict accurately.
- Hybrid Regression, ElasticNetCV, and RandomForestRegressor had moderately high R-squared scores between 46 and 55%, demonstrating reasonable prediction ability.
- SVR and KNeighborsRegressor performed the worst with very low R-squared scores of 14–15%, suggesting they were not suitable models for this dataset/problem.
- Execution times were similar to the previous table, with Hyperopt/Optuna Regression taking the longest at 300 + seconds and KNeighborsRegressor the quickest at under 0.1 s.

While the predictive performance was lower for subcutaneous compared to visceral fat, Hyperopt Regression, and Optuna Regression still achieved the best results. Their ability to optimize hyperparameters seems important for predictions, given weaker baseline model performances. Subcutaneous fat appears harder to predict but these techniques helped maximize predictive ability.

Deep analysis of Tables 5 and 6:

- **Hyperopt and Optuna regression** use sophisticated optimization techniques to fine-tune their parameters, which likely helps them achieve high accuracy. However, this parameter tuning process is computationally intensive, hence the longer execution times.
- **Hybrid regression** is designed to be fast, possibly by simplifying the model or using less computationally demanding methods. This speed comes at the cost of accuracy, as it may not capture the complexity of the data as well as the more detailed models.
- **ElasticNetCV** combines L1 and L2 regularization, which can help in dealing with multicollinearity and overfitting, leading to a model that generalizes well but isn't as accurate as the top-performing models.
- **Random Forest regressor** is an ensemble method that builds multiple decision trees and merges them to get more accurate and stable predictions. While generally robust, it may not perform as well if the data doesn't suit the assumptions made by tree-based methods.
- **SVR** works well for datasets with a clear margin of separation and is less prone to overfitting. However, it might struggle with larger datasets or those with a lot of noise, which could explain the lower accuracy.
- **Bagging regressor** also uses ensemble methods but may not be as fine-tuned as RandomForest, leading to slightly lower performance.
- **K Neighbors regressor** relies on the proximity of data points to make predictions. It's very fast but can perform poorly if the dataset has many dimensions (curse of dimensionality) or if the data isn't normalized.

**Statistical analysis: Posthoc Nemenyi test for posttreatment subcutaneous fat:**

The average ranks for each model based on the performance measures:

- Hyperopt Regression: Average Rank = 1.33
- Optuna Regression: Average Rank = 2
- Hybrid Regression: Average Rank = 5
- ElasticNetCV: Average Rank = 4.5
- RandomForestRegressor: Average Rank = 4.5
- SVR: Average Rank = 7.5
- BaggingRegressor: Average Rank = 3
- KNeighborsRegressor: Average Rank = 7.5

Since we have eight models, we can use a significance level of 0.05 to calculate the CD value. The CD value for the Nemenyi test with eight models and a significance level of 0.05 is approximately 2.536.

Now, we can compare the average ranks pairwise and check if the difference is greater than the CD value. Based on the comparisons, we can determine if there are significant differences between the models.

Comparisons:

- Hyperopt regression vs. Optuna regression: Rank difference = 0.67 (less than CD)
- Hyperopt regression vs. Hybrid regression: Rank difference = 3.67 (greater than CD)
- Hyperopt regression vs. ElasticNetCV: Rank difference = 3.17 (greater than CD)
- Hyperopt regression vs. Random Forest regressor: Rank difference = 3.17 (greater than CD)
- Hyperopt regression vs. SVR: Rank difference = 6.17 (greater than CD)
- Hyperopt regression vs. Bagging regressor: Rank difference = 0.67 (less than CD)
- Hyperopt regression vs. K Neighbors regressor: Rank difference = 6.17 (greater than CD)

Based on these comparisons, we can conclude that the Hyperopt Regression model is significantly different from the hybrid regression, ElasticNetCV, RandomForestRegressor, SVR, and K Neighbors Regressor models. However, there is no significant difference between the Hyperopt Regression model and the Optuna Regression or Bagging Regressor models.

Therefore, based on the statistical analysis using the post hoc Nemenyi test, the Hyperopt Regression model can be considered the best model among the given options.

### Feature correlations

Feature correlation is used to understand the strength and direction of the linear relationship between two variables. In the context of regression models, understanding feature correlations serves several purposes:

- **Feature Selection**: By analyzing the correlation between features and the target variable, one can identify which features have the strongest relationships with the target. This can help in selecting the most relevant features for the model, potentially improving its performance and reducing overfitting.
- **Multicollinearity Diagnosis**: High correlations between features (multicollinearity) can be problematic for some models, as it can make the model's estimates unstable and difficult to interpret. Identifying and addressing multicollinearity can lead to more reliable models.
- **Insight into Relationships**: Correlation analysis provides insights into how features are related to each other and the target variable. This can be valuable for understanding the underlying processes and for domain knowledge discovery.
- **Model Simplification**: If two features are highly correlated, it might be possible to use just one of them without losing significant predictive power, simplifying the model and reducing computation time.
- **Improving Model Accuracy**: By understanding the relationships between features, one can engineer new features that better capture the underlying patterns in the data, potentially improving the model's accuracy.

Table 7 presents the correlation coefficients between various features within a dataset. This table is structured to facilitate an understanding of how different features relate to one another. The first column lists the names of the initial features being compared, while the second column names the corresponding feature with which the first is being correlated. The third column is crucial as it contains the correlation coefficients, which quantify the strength and direction of the linear relationship between the two features. A coefficient value of one indicates a perfect positive correlation, meaning as one feature increases, the other does as well. Conversely, a value of negative one signifies a perfect negative correlation, where one feature's increase corresponds to the other's decrease. A coefficient of zero denotes the absence of any linear relationship between the pair of features.

**Table 7:** Pearson’s correlation of the features.

First feature	Second feature	Correlation
Posttreatment visceral fat	Pretreatment visceral fat	0.864
Posttreatment subcutaneous fat	Pretreatment subcutaneous fat	0.754
BMI	Weight	0.639
Height	Weight	0.464
BMI	Pretreatment visceral fat	− 0.387
BMI	Height	− 0.383
Pretreatment subcutaneous fat	Height	0.369
BMI	Posttreatment visceral fat	− 0.358
Posttreatment visceral fat	Weight	− 0.328
Posttreatment subcutaneous fat	Height	0.316
Pretreatment visceral fat	Weight	− 0.296
Pretreatment subcutaneous fat	Pretreatment visceral fat	0.275
BMI	Pretreatment subcutaneous fat	− 0.241
Posttreatment visceral fat	Pretreatment subcutaneous fat	0.218
First feature	Second feature	Correlation
BMI	Waist circumference	− 0.151
Pretreatment subcutaneous fat	Age	− 0.149
Age	Weight	− 0.132
BMI	Age	− 0.113
Posttreatment subcutaneous fat	Age	− 0.108
Posttreatment visceral fat	Waist circumference	− 0.093
Pretreatment visceral fat	Height	0.091
Posttreatment subcutaneous fat	Weight	0.09
Pretreatment subcutaneous fat	Weight	0.086
Age	Height	− 0.043
Waist circumference	Age	0.038
Posttreatment visceral fat	Age	− 0.038
Posttreatment visceral fat	Height	0.029
Pretreatment visceral fat	Age	− 0.026

Based on the Table 7:

- Posttreatment Visceral fat and Pretreatment visceral fat: There is a strong positive correlation of 0.864 between these two variables. This suggests that individuals with higher pretreatment visceral fat tend to have higher post-treatment visceral fat.
- Posttreatment Subcutaneous fat and Pretreatment subcutaneous fat: There is a strong positive correlation of 0.754 between these two variables. This implies that individuals with higher pretreatment subcutaneous fat tend to have higher posttreatment subcutaneous fat.
- BMI and Waist circumference: There is a weak negative correlation of -0.151 between BMI and waist circumference. This indicates that there is a slight tendency for individuals with higher BMI to have smaller waist circumference, but the relationship is not very strong.
- Pretreatment subcutaneous fat and Age: There is a weak negative correlation of -0.149 between pretreatment subcutaneous fat and age. This suggests that younger individuals tend to have slightly higher pretreatment subcutaneous fat levels.
- BMI and Weight: There is a moderate positive correlation of 0.639 between BMI and weight. This implies that individuals with higher weight tend to have higher BMI values.
- Height and Weight: There is a moderate positive correlation of 0.464 between height and weight. This indicates that taller individuals tend to have higher weight.

### Feature selection

The selection of feature selection^45^ algorithms is based on their ability to identify the most relevant features that contribute to the predictive power of a model, thereby improving model performance, reducing complexity, and enhancing interpretability. Each feature selection method has its own merits and is chosen for specific reasons:

- **F-value Selector**: This method selects features based on F-statistics from ANOVA tests, which evaluate the significance of each feature. It's useful for capturing linear relationships between features and the target variable.
- **Mutual Information Selector**: This technique measures the mutual dependence between variables using information gain. It is effective in capturing any kind of statistical dependency, not just linear, making it a powerful tool for feature selection.
- **RFE with Logistic Regression**: Recursive Feature Elimination (RFE) works by recursively removing the least important features based on model weights. When combined with logistic regression, it's particularly good for binary classification problems.
- **Variance Thresholding**: This method removes features whose variance doesn’t meet a certain threshold. It's a simple baseline approach to feature selection, aiming to remove features that are constant or almost constant, as they do not contribute to the model's predictive capability.
- **RFE with Random Forests**: Similar to RFE with logistic regression, but using random forests, which is an ensemble method. This combination is robust to overfitting and can capture non-linear feature interactions.
- **Feature Importance with Random Forests**: Random forests can provide a ranking of features based on their importance derived from how much they decrease the impurity of the splits. This method is useful for understanding feature contributions in complex datasets.

Table 8 shows the characteristics chosen using various feature selection approaches. The table has been analyzed and expanded as follows:

- Feature selection technique: The name of the feature selection method used to choose the features is displayed in this column.
- Selected features: The names of the features chosen by the feature selection method are displayed in this column.

**Table 8:** Feature selection techniques and the most important features.

Method	Selected features
F-value selector	BMI, waist circumference, pretreatment visceral fat, posttreatment visceral fat, pretreatment subcutaneous fat
Mutual information selector	Age, height, BMI, waist circumference, posttreatment visceral fat
RFE with logistic regression	Weight, waist circumference, pretreatment visceral fat, posttreatment visceral fat, pretreatment subcutaneous fat
Variance thresholding	Age, weight, height, BMI, waist circumference, pretreatment visceral fat, posttreatment visceral fat, pretreatment subcutaneous fat, posttreatment subcutaneous fat
RFE with random forests	BMI, waist circumference, pretreatment visceral fat, pretreatment subcutaneous fat, posttreatment subcutaneous fat
Feature importance with random forests	Waist circumference, pretreatment visceral fat, pretreatment subcutaneous fat, BMI, age

Table 7 presents the correlation coefficients between various features within a dataset. This table is structured to facilitate an understanding of how different features relate to one another. The first column lists the names of the initial features being compared, while the second column names the corresponding feature with which the first is being correlated. The third column is crucial as it contains the correlation coefficients, which quantify the strength and direction of the linear relationship between the two features. A coefficient value of one indicates a perfect positive correlation, meaning as one feature increases, the other does as well. Conversely, a value of negative one signifies a perfect negative correlation, where one feature's increase corresponds to the other's decrease. A coefficient of zero denotes the absence of any linear relationship between the pair of features.

Based on the Table 8:

- F-value selector and RFE with logistic regression selected the same 5 features—BMI, waist circumference, pre-treatment visceral fat, post-treatment visceral fat, and pre-treatment subcutaneous fat. These methods focused on the most statistically significant predictive features.
- Variance thresholding selected all 9 features, meaning they all provided some unique information. However, it does not rank the importance.
- Mutual information selector and RFE with random forests selected very similar features with some differences, showing agreement between these information-theory and ensemble-based techniques.
- Feature importance with random forests directly ranks feature importance, showing waist circumference, pre-treatment visceral fat, and subcutaneous fat as the top predictors along with BMI and age.

Based on the consistency between the F-value selector, RFE with logistic regression, and feature importance with random forests, we recommend using the feature importance with random forests approach. It directly ranks useful feature importance, and the top 5 features it selected are consistent with the other top-performing methods. Considering it incorporates an ensemble technique rather than just statistical testing, it may provide a more robust ranking of predictive power. The top features it identified are also clinically interpretable risk factors.

### Hyperparameter tuning

The meticulous tuning of hyperparameters is crucial to the performance of machine learning models. These adjustable parameters govern the model's structure, the learning process, and the strategy for optimization. Tables 9 and 10 provide the hyperparameter values selected for this investigation, ensuring that our results are transparent and can be replicated. These tables offer an exhaustive look at the principal parameters that influenced the training and behavior of the model, granting insight into the experimental framework and identifying opportunities for further research or refinement.

**Table 9:** Hyperparameter optimization with hyperopt.

Hyperparameter	Value range	Description
n_estimators	100–1000 (step 100)	Determines the number of trees in the random forest
max_depth	5–30	Controls the maximum depth for each tree
min_samples_split	2–20	Sets the minimum samples required to split an internal node
min_samples_leaf	1–10	Defines the minimum samples required at a leaf node
random_state	42	Ensures consistent results across multiple runs

**Table 10:** Optuna hyperparameter optimization for Random Forest regression—selected best hyperparameters.

Hyperparameter	Best value	Description
n_estimators	600	The optimal number of trees in the forest for enhanced performance
max_depth	17	The ideal maximum depth of the tree to prevent overfitting
min_samples_split	5	The optimal minimum number of samples required for splitting an internal node
min_samples_leaf	2	The ideal minimum number of samples required at a leaf node

### Discussion

Cavitation has occurred. Ultrasonic waves generate a series of contraction and expansion cycles, which apply positive and negative pressure. This pushing and pulling force might cause fat cells to fracture. As well as enhancing body contouring in medical and aesthetic therapies^46^. The ability to understand the effects of cavitation on abdominal fat dynamics is crucial for developing effective treatment strategies and advancing non-invasive fat reduction techniques. In this study, we employed two state-of-the-art hyperparameter optimization frameworks, Hyperopt and Optuna, to optimize fat prediction models and minimize the uncertainty associated with these models.

By leveraging the capabilities of Hyperopt and Optuna, we aimed to unlock the full potential of fat prediction models and enable accurate predictions of abdominal fat dynamics in the context of cavitation’s impact. Our experiments utilized a comprehensive dataset containing measurements of abdominal fat and cavitation parameters. We employed various regression models, including Hyperopt Regression and Optuna Regression, and evaluated their performance using metrics such as mean squared error, mean absolute error, and R-squared score.

The results demonstrated that our approach using Hyperopt and Optuna regression models achieved high R-squared scores for posttreatment visceral fat and posttreatment subcutaneous fat predictions. These high scores indicate that our models accurately captured the variations in fat dynamics influenced by cavitation. The findings highlight the effectiveness of our approach in predicting fat dynamics and provide valuable insights for clinicians, researchers, and individuals seeking non-invasive fat reduction solutions.

Additionally, we explored feature selection techniques to identify the most important features contributing to the fat prediction models. The selected features varied across the employed techniques, emphasizing the significance of factors such as BMI, waist circumference, pretreatment visceral fat, posttreatment visceral fat, and pretreatment subcutaneous fat in predicting fat dynamics. These findings contribute to our understanding of the factors influencing the effectiveness of cavitation treatments and provide valuable insights for treatment customization and optimization.

## Limitations

Despite the significant contributions and promising results of our study on predicting abdominal fat dynamics in the context of cavitation treatments, several limitations should be acknowledged:

1. **Limited Sample Size:** The study utilized a comprehensive dataset; however, the sample size may still be relatively small. A larger sample size would enhance the statistical power and generalizability of the findings. The limited sample size may also restrict the exploration of potential subgroups or variations within the population.
2. **Restricted to Cavitation Treatments:** The study exclusively focused on cavitation treatments for fat reduction. While this provides valuable insights into the effects of cavitation, it limits the generalizability of the findings to other non-invasive fat reduction techniques or combinations of treatments.
3. **Feature selection:** While feature selection techniques identified important factors, other relevant features might not have been included in the analysis. Further investigation into additional features related to patient health, treatment parameters, or cavitation device specifics could improve model accuracy.
4. **Model interpretability:** The study primarily focused on the R-squared score and other performance metrics for model selection. While Random Forests offer some insights through feature importance, a deeper exploration of model interpretability techniques would be valuable. Understanding how the model arrives at its predictions could provide valuable clinical insights into the mechanisms underlying fat reduction with cavitation.
5. **Limited exploration of alternative models:** The study focused on Hyperopt and Optuna for hyperparameter optimization with Random Forest Regression. Exploring other machine learning models or hyperparameter optimization techniques might yield even better performance or uncover new insights into the data.

## Conclusions and future directions

This study focused on the application of cavitation in reducing abdominal fat and improving body contouring. By utilizing state-of-the-art hyperparameter optimization techniques, Hyperopt and Optuna, the aim was to enhance the accuracy and predictive capabilities of fat prediction models in the context of cavitation treatments. Through comprehensive experiments using a dataset encompassing measurements of abdominal fat and cavitation parameters, the performance of various regression models was evaluated. The Hyperopt Regression and Optuna Regression models exhibited high R-squared scores of 94.12% and 94.11% for posttreatment visceral fat prediction, and 71.15% and 70.48% for posttreatment subcutaneous fat prediction, respectively. These results demonstrate the effectiveness of the approach in accurately predicting fat dynamics following cavitation treatments. The findings of this study have important implications for the advancement of non-invasive fat reduction techniques. By improving the understanding of how cavitation influences abdominal fat dynamics, the development of more effective treatment strategies can be guided. These insights benefit both researchers and practitioners in the field of fat reduction, as predictions of treatment effects are two key components in modern medicine and personalized healthcare. Addressing these limitations in future research will further strengthen the understanding of cavitation's impact on abdominal fat dynamics and refine the predictive models. By leveraging advanced optimization frameworks and robust regression models, a step has been taken toward unlocking the full potential of fat prediction models and paving the way for future-focused predictions in the field of fat reduction.

The results and analyses presented in this study provide a solid foundation for future research in the field of regression machine learning techniques. The following areas have been identified for further exploration:

1. Model Generalization: Future studies should focus on validating the models with external datasets to assess their generalizability. This will help determine the robustness of the models across different populations and conditions.
2. Algorithmic Improvements: There is scope for developing more efficient algorithms for hyperparameter optimization that reduce computational time without compromising the predictive performance of the models.
3. Interpretability Enhancements: Efforts should be made to improve the interpretability of complex models, such as Hyperopt Regression and Optuna Regression, to make the results more accessible to practitioners.
4. Real-time Predictions: Investigate the feasibility of these models for real-time prediction scenarios, where execution time is critical, by optimizing the models for faster performance.
5. Deep Learning Approaches: Investigate the application of deep learning models for the regression tasks, as they may capture non-linear relationships and interactions more effectively.
6. Clinical Applications: Explore the clinical implications of the model's predictions and how they can be integrated into healthcare practice for better patient outcomes.
7. Software Development: Develop user-friendly software tools that incorporate the best-performing models, making them accessible to researchers and clinicians without a background in machine learning.
8. Comparative Analysis: Comparing the performance of different optimization frameworks^47,48^ and regression models can help identify the most effective approaches for fat prediction in the context of cavitation treatments. Comparative studies can guide researchers and practitioners in selecting the optimal techniques for their specific needs.

## Data Availability

The dataset and code used in this study are public and all test data are available at this portal (https://github.com/tarekhemdan/Cavitation_ML).
